# The alternative for being an alternative

**DOI:** 10.1186/s42155-024-00488-w

**Published:** 2024-10-11

**Authors:** Jim A. Reekers

**Affiliations:** Emeritus Professor of Interventional Radiology, Amsterdam UMC, University of Amsterdam, Meibergdreef 9, Amsterdam, The Netherlands

Visiting the last annual meeting of CIRSE in Lisbon showed me the huge leaps IR has made in the last decades. It was one of the best scientific meetings we have ever had with an overwhelming attendance. There was a spirit of excitement to see which new territories we have entered. As editor in chief of the CIRSE endovascular journal I will skip the great achievements in the field of oncology which are now the third pillar of oncology treatment and concentrate on the endovascular treatments which have also been pilling up during the last decades. Next to the joy of all this excitement I also feel the urge to be a devils advocate by putting this all in perspective. We should not kiss ourselves to sleep with complacency. We have now much more to offer to patients than two decades ago but the world outside has still no desire to join our party. I tried to make that point with my previous editorial : Getting it right is better than being right, right [1]? Just to note that our new treatments have still not really changed medical thinking in many hospitals is not a way forward. Yes, being an independent specialty with direct referral could be a part of the solution. But as has been shown in the US with the double certification, it will not end turf battles and specialty centred treatment choices. It is time for a new IR mindset.

Surgery as we know it today goes back to the early nineteenth century and is therefore already 200 years old. Some mark the first transvaginal hysterectomy in 1828 by James Blundell as the start of modern surgery. Soon to be followed by Charles Clay with the first abdominal hysterectomy in 1843. Surgery has been the only invasive solution for many medical problems for centuries. However, surgery has also never been challenged during al this time and has therefore not felt the urge to innovate. Improvements in surgery outcomes should mainly be contributed to technological progress in anaesthesiology and intensive care medicine but not surgical innovations. The problem with such an ancient profession is that over time you become a panacea like when you are a hammer everything looks like a nail. Lack of competition or alternative treatments lulls the need to innovate to sleep. In 1963 Charles Dotter challenged vascular surgery by introducing angioplasty, which was 10 years later refined as balloon angioplasty. For a decade Dotter was banned from the American medical stage as being a dangerous alternative and a lunatic person. From that early start of IR, we were able to develop new treatments like thrombectomy, thrombolysis, embolization and stenting. At the end of that century, in a period of less than 40 years, we were able to offer our broad spectrum of new treatments based on new IR technologies as an alternative to vascular surgical treatments. Gradually, those patients not fit for the real treatment, being off course surgery, were more and more offered the IR alternative. In 1990 Juan Parodi, a surgeon and Julio Palmaz a trained Interventional radiologist, performed the first successful endovascular abdominal aortic aneurysm (AAA) repair in Buenos Aires [2]. It took a few years before surgeons realized that this new technology based on the IR invented stent technology could be an existential threat to surgery when performed by IR. This was no longer an alternative treatment by some marginal group of radiologists this was an innovation that could not be overlooked. Within no time the technology was renamed in Endovascular surgery and claimed to be invented by the surgeons Juan Parodi and in Russia Nicolai Volodos, crossing out Palmaz from the historical record. Like they tried to do with Dotter. The historical facts are however clear as it was Parodi and Palmaz who did the first successful endograft in 1990 followed Volodos in 1993. No IR protested loudly or tried to claim endograft treatment with stents and a percutaneous technique as belonging to the field of both specialties, surgery and IR. We than missed a great opportunity to emancipate from an alternative treatment specialty to a full-blown treatment specialty. This mistake has been haunting us for the decades. The stigma of being radiologists/imagers with an alternative treatment was after that missed opportunity engraved in the image of IR. Today we have developed many more treatments but we still can only offer them by knocking on the backdoor of those specialties who claim the birthrights and control over the patients. Fibroid embolisation is not an alternative to hysterectomy it is, if you look at the literature, the first-choice treatment and should therefore be called fibroid treatment from now on. The same is true for prostate embolisation, percutaneous treatment of CLTI, thrombolysis, thrombectomy for venous disease and acute pulmonary embolism, MSK embolisation and many more. We have to stop promoting these treatments as an alternative to long existing treatments and stop being afraid of telling the other medical specialists that they do not have the best treatment option for the patient available anymore. And we should tell patients that they are deprived of their best option. Stop competing with surgical endpoints but advertise the specific IR endpoints which are always more patient friendly, as the best contemporary option. For fibroid embolisation preservation of the uterus is a better endpoint from a patient perspective than total absence of menorrhagia. Improved micturition after PAE is better than diapers, impotence and retrograde ejaculation after TURP. Amputation free survival (AFS) after endo treatment is better than a patent bypass. Carotid stenting has no sympathetic nerve damage and an equal outcome looking at major stroke and dead as endpoint. We should start scientific studies to find the best endpoints from a patient perspective and not try to compete with old self-centred surgical endpoint as procedural success. It is time to finally set aside our modesty and to start telling our own story. This is the only alternative for not staying an alternative. There is no reason to be shy and not to be proud of all our great IR achievements.
